# Nasal immature teratoma in an elderly patient: Clinicopathological and epigenetic analogies with central nervous system counterparts, alongside genomic divergences

**DOI:** 10.1111/neup.13008

**Published:** 2024-10-02

**Authors:** Shintaro Inoue, Hirokazu Takami, Shota Tanaka, Masashi Nomura, Shunsaku Takayanagi, Yuki Saito, Shu Kikuta, Kenji Kondo, Reiko Matsuura, Masako Ikemura, Sho Yamazawa, Masao Matsutani, Ryo Nishikawa, Yuko Matsushita, Koichi Ichimura, Nobuhito Saito

**Affiliations:** ^1^ Department of Neurosurgery The University of Tokyo Hospital Tokyo Japan; ^2^ Department Otolaryngology, Head and Neck Surgery The University of Tokyo Hospital Tokyo Japan; ^3^ Department Pathology The University of Tokyo Hospital Tokyo Japan; ^4^ Department of Neuro‐Oncology/Neurosurgery Saitama Medical University International Medical Center Saitama Japan; ^5^ Department of Brain Disease Translational Research Juntendo University Faculty of Medicine Tokyo Japan

**Keywords:** brain tumor, germ cell tumor, immature teratoma, intranasal tumor teratoma

## Abstract

Germ cell tumors (GCTs) are categorized as gonadal or extra‐gonadal, based on the origin. Extra‐gonadal GCTs predominantly manifest within the central nervous system (CNS), mediastinum, retroperitoneum, and sacrococcygeal region. These malignancies are most frequently diagnosed in the pediatric, adolescent, and young adult demographics. Incidences of GCT within the nasal cavity are notably scarce, with only six cases documented. This report details the case of a 70‐year‐old man who presented with a left nasal mass ultimately diagnosed as immature teratoma. A remarkable aspect of this case was the detection of SMARCA4 (BRG1) loss through immunohistochemical analysis. In addition, methylation profiling aligned this case with CNS GCTs, specifically those classified as non‐germinomatous GCTs. This molecular characterization informed a tailored therapeutic strategy incorporating carboplatin and etoposide, alongside localized irradiation. This individualized treatment regimen achieved favorable outcomes, with the patient remaining recurrence free for over three years. This highlights the need for precise therapeutic approaches in the management of extragonadal GCTs, particularly those arising in atypical anatomical locations. The present case accentuates the significance of thorough diagnostic evaluations and customized treatment plans for rare GCT presentations. Further empirical and clinical investigations are warranted to enhance our understanding of and refine therapeutic protocols for such exceptional cases.

## INTRODUCTION

Germ cell tumors (GCTs) are postulated to derive from primordial germ cells (PGCs) and predominantly manifest within gonadal tissues such as the testes and ovaries during the second and third decades of life.^1^, ^2^ GCTs that arise external to these canonical sites, comprising less than 5% of cases, are referred to as extragonadal GCTs. These neoplasms characteristically emerge along the midline of the body, notably within the central nervous system (CNS), mediastinum, retroperitoneum, and sacrococcygeal area.^3^ CNS GCTs are primarily diagnosed in children, adolescents, and young adults, with a peak incidence between 12 and 16 years old.^4^ Mediastinal GCTs are most frequently identified among individuals in their twenties and thirties, retroperitoneal GCTs have a median age at diagnosis of around 40 years, and sacrococcygeal GCTs are predominantly observed in newborns and young children.^5^, ^6^ The histological landscape of GCTs is varied, encompassing subtypes such as germinomas/seminomas/dysgerminomas, mature and immature teratomas, teratomas with somatic‐type malignancy, yolk sac tumors, choriocarcinomas, and embryonal carcinomas, all classified under the framework defined by the World Health Organization.^7^

Recent molecular analyses have elucidated characteristic genetic aberrations within GCTs, including a recurrent gain of chromosome 12p, alongside frequent mutations within the RTK, MAPK, and PI3K signaling pathways.^8^, ^9^, ^10^, ^11^ Intriguingly, methylation patterns differ markedly between germinomas/seminomas and non‐germinomas/non‐seminomas, with the former group exhibiting exceptionally low methylation levels across chromosomes, akin to those of PGCs. Notably, germinomas and seminomas share methylation profiles, suggesting a similarity in biological underpinnings between CNS and testicular variants.^12^

Extragonadal GCTs, excluding those arising within the CNS, mediastinum, retroperitoneum, and sacrococcygeal regions, are exceedingly rare. Specifically, GCTs occurring within the nasal cavity have been documented in only a very small number of case reports. The clinical presentation, underlying biology, and optimal treatment strategies for these tumors thus remain poorly defined. This study introduces a rare case of intranasal immature teratoma with detailed clinical characteristics and results of histopathological analyses and biological profiling in juxtaposition with CNS counterparts. This comprehensive examination facilitated the adoption of a treatment strategy extrapolated from that used for CNS GCTs, ultimately achieving sustained tumor control and a favorable prognosis.

## CLINICAL SUMMARY

A 70‐year‐old man presented with persistent discomfort localized within the left nasal cavity that progressively worsened over a two‐month period. Initial evaluation at a local otorhinolaryngological clinic revealed the presence of a mass lesion within the left nasal cavity, prompting referral to the otorhinolaryngology department of our institution for comprehensive assessment and formulation of a treatment strategy. Initial physical examination, aside from revealing nasal congestion, failed to disclose any significant abnormalities. The patient had no contributory medical history.

**Fig 1:**
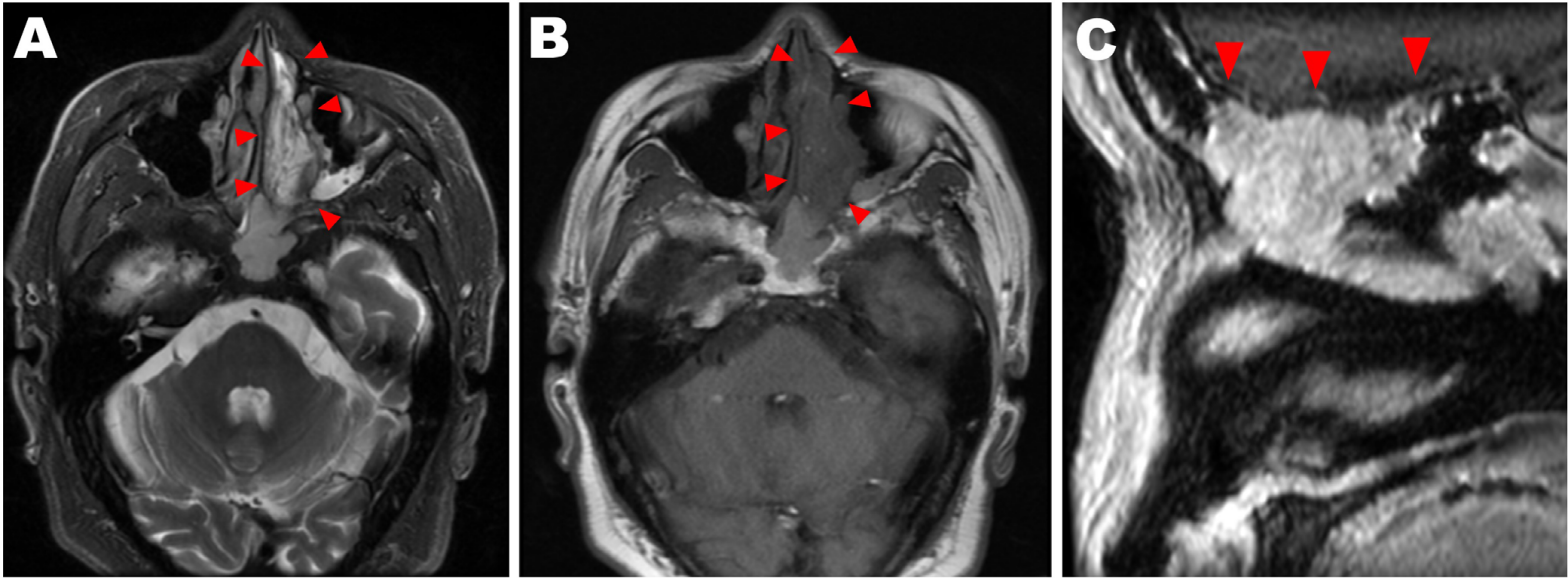
MRI at initial presentation. The lesion shows hyperintensity on T2‐weighted imaging (A), hypointensity on T1‐weighted imaging (B), and no extension beyond the anterior skull base to the intracranial space (C).

Magnetic resonance imaging (MRI) of the head delineated a mass approximately 5 cm in length spanning from the left nasal cavity to the posterior segment of the paranasal sinuses. The lesion exhibited heterogeneity in T2 signal intensity, with nodular formations and membranous structures accompanied by diffusion restriction (Fig. 1). Notably, no evidence of tumor invasion into the cranial vault or signs of osseous erosion were identified. Fluorodeoxyglucose‐positron emission tomography identified significant radiotracer uptake within the lesion (SUVmax: approximately 9), albeit without indications of any other systemic lesion. CT covering the area from the neck to the pelvis revealed no additional lesions. The constellation of imaging findings and clinical trajectory led to differential diagnoses encompassing benign vascular entities, such as capillary hemangioma or pyogenic granuloma, with consideration also given to inverted papilloma based on radiological interpretation. The lack of potential for spontaneous resolution through conservative management underscored the need to recommend surgical excision. An explanation of procedures and perceived risks and benefits of the recommended surgical approach was provided, and the patient provided informed consent to proceed.

The therapeutic intervention entailed an endoscopic surgical resection of the neoplasm situated within the left nasal cavity. Gross total resection was achieved, with meticulous sparing of adjacent healthy tissue.

## PATHOLOGICAL FINDINGS

**Fig 2:**
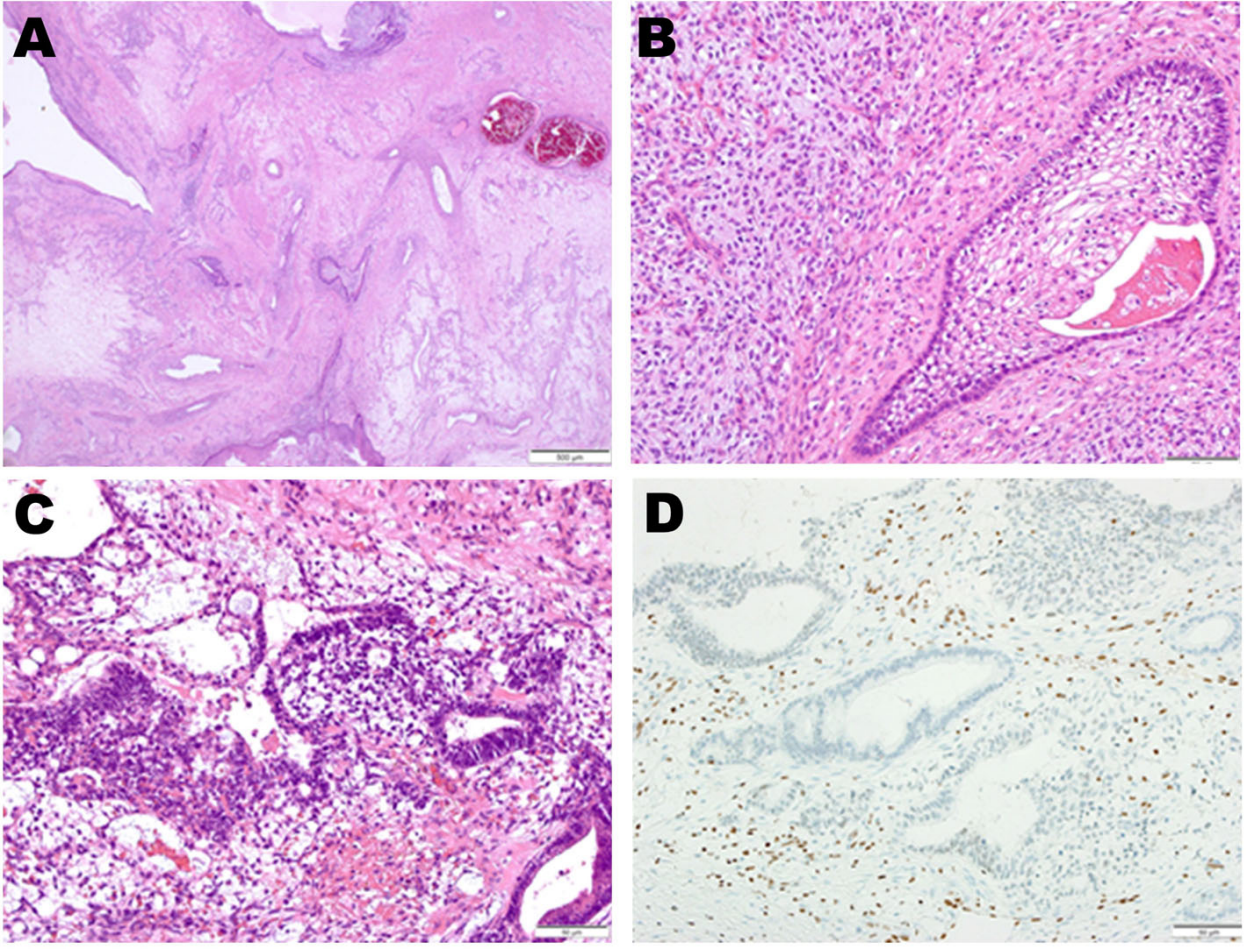
(A) Hematoxylin and eosin (HE) staining reveals a histological panorama in which epithelial tissue, mostly squamous, is embedded in abundant edematous or myxoid stroma. (B) Squamous epithelium with clear cells is surrounded by myxoid stroma, with both components displaying primitive characteristics and minimal cytological atypia. (C) Atypical cells featuring round hyperchromatic nuclei, and a high nuclear‐to‐cytoplasmic (N/C) ratio form a complex primitive glandular or neuroepithelial‐like structure. (D) Immunohistochemically, epithelial cells exhibit complete loss of BRG1 (SMARCA4) alongside with a subset of interstitial cells. Endothelial and immune cells retain BRG1 expression, serving as a positive control for staining.

Pathologically, a small amount of immature epithelial tissue was embedded within a large area of granulation‐like fibrous stroma. Atypical cells with round, hyperchromatic nuclei and a high nuclear‐to‐cytoplasmic (N/C) ratio formed complex, primitive glandular, or neuroepithelial‐like structures. Both keratinizing and non‐keratinizing squamous epithelium, without cytological atypia, wrere observed, with some parts displaying an immature appearance with clear cytoplasm. A minor amount of serous or mucinous epithelium was also identified. The surrounding stroma comprised fibroblast or myofibroblast‐like spindle cells with minimal cytological atypia and numerous capillary vessels. Cell density was low, and mitotic figures were exceptionally rare. Portions of the stroma exhibited primitive features with myxoid or edematous changes. Vessels with irregular wall thickening and smooth muscle proliferation were present, while no bone or cartilage tissue was detected (Fig. 2A–C).

Immunohistochemically, the immature glandular or neuroepithelial‐like tissues were partially positive for SALL4, glypican 3, or alpha‐fetoprotein. Of particular note was the complete loss of BRG1 (SMARCA4) expression in epithelial cells, along with a portion of interstitial cells. In contrast, endothelial and immune cells retained BRG1 expression, serving as a positive control for staining (Fig. 2D). Expressions of BRM (SMARCA2) and INI1 (SMARCB1) were maintained across all cells, and p53 displayed a wild‐type staining pattern, with only rare cells testing positive.

Despite not adhering to classical clinicopathological characteristics, immature teratoma was diagnosed, as no other diagnosis provided a more fitting description. Of note, no other GCT components, such as germinoma, yolk sac tumor, choriocarcinoma, or embryonal carcinoma, were found in the exhaustive examination of the entire specimen. Furthermore, the blastemal component, characterized by densely proliferating cells with a high N/C ratio, was not observed. Components corroborating teratocarcinosarcoma were also not found.

## MOLECULAR GENETIC FINDINGS

**Fig 3:**
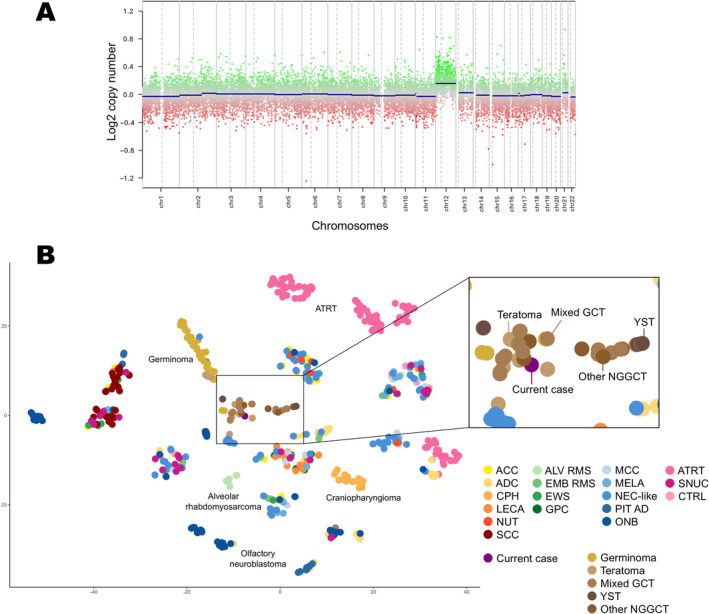
(A) Copy‐number analysis shows a chromosomal aberration characterized by the gain of chromosome 12p, a genetic alteration frequently associated with gonadal, and extra‐gonadal germ cell tumors. (B) Using t‐distributed stochastic neighbor embedding (t‐SNE) plots for comparative genomic analysis, the tumor exhibits a clustering pattern aligning closely with those of non‐germinomatous germ cell tumors, distinguishing it within a diverse spectrum of CNS and sinonasal tumor entities. ACC, sinonasal adenoid cystic carcinoma; ADC, sinonasal adenocarcinoma; ALV RMS, alveolar rhabdomyosarcoma; ATRT, adult pituitary atypical rhabdoid/teratoid tumor; CPH, craniopharyngioma; CTRL, normal sinonasal control tissue; EWS, Ewing's sarcoma; EMB RMS, embryonal rhabdomyosarcoma; GCT, germ cell tumor; GPC, sinonasal glomangiopericytoma; LECA, lymphoepithelial carcinoma; MCC, Merkel‐cell carcinoma; MELA, sinonasal mucosal melanoma; NEC‐like, sinonasal neuroendocrine carcinoma‐like tumors; NUT NUT, midline carcinoma; ONB, olfactory neuroblastoma; PDCA, sinonasal poorly differentiated carcinoma; PIT AD, pituitary adenoma; SCC, sinonasal squamous cell carcinoma; SNUC, sinonasal undifferentiated carcinoma.; NGGCT, non‐germinomatous germ cell tumor; YST, yolk sac tumor.

To corroborate the pathological diagnosis and delve deeper into the molecular underpinnings of the tumor, comprehensive analyses involving methylation profile and copy‐number variations were conducted. DNA was extracted from the formalin‐fixed paraffin‐embedded tumor specimen using a QIAamp DNA Mini Kit (Qiagen, Tokyo, Japan), following the protocol from the manufacturer. Extracted DNA was analyzed using the Infinium MethylationEPIC Kit version 1.0 (Illumina, Tokyo, Japan), again adhering to the guidelines from the manufacturer. Methylation profiling leveraged the DKFZ classifier developed by the German Cancer Research Center (DKFZ)/University Hospital Heidelberg/German Consortium for Translational Cancer Research (DKTK) (accessible at molecularneuropathology.org), using their Brain Tumor Classifier version 12.5. This analytical tool categorized the case within the “Teratoma” methylation class, assigning a calibrated score of 0.84632. Analysis of raw signal intensities derived from IDAT data files was executed using the minfi Bioconductor package (version 1.44.0; R version 4.2.2), facilitating the calculation of β values as previously described.^13^ The investigation into copy number variations was conducted through the Conumee package (version 1.9.0;), which revealed a gain in chromosome 12 (Fig. 3A). For exhaustive examination, an unsupervised nonlinear dimension reduction technique was employed, specifically t‐distributed stochastic neighbor embedding (t‐SNE). This involved the selection of the 20 000 most variable probes from the reference cohort of 82 CNS GCTs^8^ (GSE70783) and reference samples of representative CNS and sinonasal tumors (GSE109381, GSE189778),^12^, ^14^, ^15^, ^16^ predicated on standard deviations. The resultant analyses were graphically represented using the Rtsne package (version 0.15;). Within the t‐SNE plots, tumor positioning was closely aligned with non‐germinomatous GCTs (Fig. 3B), thus providing invaluable insights into the biological landscape of the tumor.

### Treatment and follow‐up

In the aftermath of deliberative sessions by the Cancer Board, a consensus was reached to embark on adjuvant therapy aligning with the treatment protocol for intracranial immature teratomas. The regimen initiated comprised three cycles of the CARE protocol, incorporating carboplatin at a dosage of 450 mg/m^2^ and etoposide at 150 mg/m^2^, in conjunction with intensity modulated radiation therapy (IMRT) delivering a total dose of 54 Gy across 30 fractions. Given the patient's advanced age, it was prudently decided to commence CARE therapy at 60% of the standard dosage. The initial cycle was well tolerated without any significant adverse effects, prompting an escalation to 70% of the dosage for the second cycle. Unfortunately, this adjustment precipitated grade 3 pancytopenia, necessitating a reversion to the initial dosing parameters for the third cycle, thereby concluding the chemotherapy phase of treatment. Complications arising from IMRT were limited to grade 2 radiation dermatitis/mucositis, which was effectively managed through topical interventions. The adjuvant therapy was executed as planned, and at the three‐year mark post‐treatment, the patient remains free of recurrence, attesting to the efficacy and strategic modulation of the therapeutic approach.

### Literature review

A comprehensive review of the literature was conducted, using PubMed as the primary source of scholarly articles, to collate data on intranasal GCTs documented between 1950 and 2024. The search strategy incorporated a nuanced combination of keywords such as “nasal”, “intranasal”, and “germ cell tumor”. This search identified six case reports, delineating a spectrum of presentations ranging from purely intranasal tumors to intracranial tumors with notable extension into the nasal cavity (Table 1).

**Table 1:** Previous reports of intranasal germ cell tumors

	Previous reports of pure germinoma of the medulla oblongata						
Author (year)	Age/Sex	Histological diagnosis	location	Surgical treatment	Adjuvant therapy	Follow‐up period	Survival
R.D Chatterjee et al. (1999)	4 months/F	‐	Right nostril	‐	‐	‐	Not described
Titus S Ibekwe et al. (2007)	18 months/F	Mature teratoma	Right nasal cavity	GTR	‐	31 months	Alive
Fang Guo et al. (2022)	28 years/F	Immature teratoma with mature elements	Left submandibular lesion	biopsy	BEP therapy	‐	Dead (pulmonary infection)
Subhro Ganguly et al. (2018)	14 years/M	Immature teratoma	Left nasal cavity	GTR	BEP therapy	2 years	Alive
Carmen Georgiu et al. (2016)	35 years/M	Immature teratoma	basal part of the left frontal lobe with extension to parasal sinus	GTR (two times: for the initial lesion and the relapse lesion)	BEP therapy(initial) TIP therapy with radiotherapy and gmma‐kinfe surgery (relapse, not otherwise described)	6 years	Alive
Israel et al. (2016)	27 years/F	Immature teratoma	Right nasal cavity with extension to right frontal lobe	GTR with second‐staged surgery (subfrontal approach+transnasal approach)	‐	6 months	Alive
Present case	70/M	Immature teratoma	Left nasal cavity	PR	CARE therapy with IMRT (54 Gy/30 Fr.)	5 months	Alive

BEP, bleomycin, etoposide, and cisplatin; CARE, carboplatin, etoposide; GTR, gross total resection; IMRT, intensity modulated radiation therapy; PR, partial resection; STR, subtotal resection; TIP, paclitaxel, ifosfamide, and cisplatin therapy.

The first case involved an 18‐month‐old girl with histologically verified mature teratoma originating from the nasal septum, exhibiting extensive involvement of the paranasal sinuses and nasal cavity, mirroring aspects observed in the current investigation.^17^ The second case detailed a four‐month‐old girl presenting with a pedunculated mass projecting from a nostril since birth. The firm, non‐tender mass measured 5 cm in length and was confined to the intranasal space, initially presumed to be teratoma, although histopathological confirmation was lacking due to the absence of parental consent.^18^ The third narrative encompassed a 28‐year‐old woman initially presenting with metastasis to lymph nodes in the left submandibular region, later diagnosed with intranasal immature teratoma from pathological assessment. Despite the regimen of bleomycin, etoposide, and cisplatin (BEP) proving efficacious in tumor management, the patient succumbed to a respiratory infection 11 months post‐diagnosis.^19^ The fourth account detailed a 14‐year‐old boy with symptoms of nasal obstruction and rhinorrhea, found to have a polypoidal mass in the nasal cavity. Diagnostic imaging revealed a soft tissue lesion penetrating the cribriform plate. Endoscopic sinus surgery elucidated intracranial extradural extension, with successful resection and repair of a cerebrospinal leak. Pathology confirmed immature teratoma. Adjuvant BEP chemotherapy achieved a two‐year recurrence‐free interval.^20^ The fifth case involved a 35‐year‐old man presenting with seizures. Imaging suggested an intracranial tumor extending from the anterior skull base into the sinuses. Following surgical excision, histopathological examination revealed immature teratoma.^21^ The patient received BEP chemotherapy, followed by a relapse treatment regimen comprising paclitaxel, ifosfamide, and cisplatin, achieving six years of recurrence‐free survival. The sixth case detailed a 27‐year‐old woman presenting with headache and nasal congestion, in which imaging revealed intracranial and intranasal masses. Surgical intervention for both components affirmed pathological findings consistent with immature teratoma, and the patient maintained recurrence‐free status for six months post‐surgery, without the need for adjuvant chemotherapy or irradiation.^22^

## DISCUSSION

The most common manifestation of CNS GCT is germinoma, which predominantly localizes to midline cerebral structures, specifically the pineal gland and neurohypophysis, in descending order of frequency.^4^, ^23^, ^24^ Among non‐germinomatous GCTs, immature teratoma is a notable histological variant. This subtype may present either as an isolated histopathological entity, particularly in patients under six years old, or as a component within mixed GCTs across other age demographics.^25^ Given the hypothesis that progenitor cells of GCTs, PGCs, are destined to differentiate into gonadal cells within the testes and ovaries, GCTs arising outside these gonadal sites are classified as extragonadal GCTs, with the CNS being a primary extragonadal site. Whether gonadal or extragonadal, GCTs are predominantly found in pediatric and young adult populations, attributed to the persistence of PGCs by evading apoptosis and potentially mis‐migrating, acquiring genomic alterations in the process.^26^ Intriguingly, the case under discussion involves an elderly patient diagnosed with primary, solitary GCT within the nasal cavity, an exceedingly rare occurrence that merits particular attention. This clinical case failed to evaluate the tumor markers characteristic of GCTs, such as human chorionic gonadotropin (HCG) and alpha‐fetoprotein (AFP), which could have assisted in differentiating the diagnosis preoperatively.

The ideal therapeutic approach for CNS immature teratoma remains under debate, oscillating between exclusive surgical resection and a combination of resection, chemotherapy, and irradiation.^27^, ^28^, ^29^, ^30^ This quandary extends to the management of GCTs situated in unconventional locations, such as the intranasal space, where their rarity compounds the complexity of treatment decision‐making. The histopathological features observed in this case partly align with those typically associated with immature teratomas of the CNS. It was predominantly composed of immature squamous epithelium, although the germ layer from which this component is derived remains unclear. Further, methylation and copy‐number analyses revealed a methylation profile and characteristic gain of chromosome 12p, respectively, mirroring findings in CNS teratoma.^8^ These genetic markers suggest that intranasal teratomas share significant biological parallels with their CNS counterparts, bolstering the rationale for applying CNS GCT treatment protocols to this case.^31^ BRG1 loss on immunohistochemistry suggested the presence of genetic alterations in *SMARCA4*. *SMARCA4* functions as an integral component of the SWI/SNF chromatin remodeling complex, a pivotal regulator of cellular differentiation during development, and exerts suppressive influences on the process of tumorigenesis.^32^ This particular gene alteration, often delineated beyond the scope of CNS GCTs, is notably associated with atypical teratoid/rhabdoid tumors.^33^, ^34^ The presence of *SMARCA4* alterations or BRG1 loss in immunohistochemistry has not been reported in teratomas. Sinonasal carcinoma with SMARCA4‐deficiency have been documented as a rare entity that appears to show a much more aggressive clinical course.^35^ Sinonasal teratocarcinosarcoma, another differential diagnosis, is also known as a clinically aggressive tumor, but the absence of atypical cells representing carcinoma or sarcoma in the current case made this diagnosis unlikely. Furthermore, no such high‐grade or undifferentiated components were observed in this case. However, the occurrence at an older age in this case was more consistent with such a diagnosis. In addition, teratocarcinosarcoma has been reported to lack a 12p gain, which could allow differentiation between teratoma and teratocarcinosarcoma.^36^ Despite the absence of 19p chromosomal loss or specific segment deletions as determined by copy‐number analysis, evaluation of mutations was hindered by the insufficient quantity and quality of DNA available in this instance. However, alterations in the *SMARCA4* gene likely played a pivotal role in the emergence of this neoplasm.

Adjustments to treatment intensity were imperative, considering the advanced age of the patient, but the tailored approach proved efficacious. The patient has remained free of both local and distant recurrences for over three years—a noteworthy achievement given the typical pattern of non‐germinomatous GCT recurrence. One report described three‐year progression‐free and overall survival rates for immature teratoma of 65% and 56%, respectively.^29^ While the accumulation of additional cases is eagerly awaited, the genomic insights and therapeutic outcomes documented here enrich the existing knowledge base for these rare entities. By demonstrating the feasibility of applying intracranial germ cell tumor treatment strategies to nasal germ cell tumors, this case contributes valuable perspectives to the evolving discourse on GCT management.

### Conclusion

An exceedingly uncommon instance of immature teratoma arising within the nasal cavity has been documented, complete with exposition of the genomic profile and therapeutic strategy. This particular intranasal GCT demonstrated a methylation profile and genomic alterations akin to those observed in CNS GCTs, implying a conserved biological essence irrespective of the anatomical site of emergence. The congruence in these biological underpinnings lends credence to the efficacy of treatment regimens comprising platinum‐based chemotherapy alongside focal radiation therapy, which proved instrumental in managing the tumor in this distinctive case. The imperative for further scholarly inquiry and accrual of clinical insights remains pronounced for such extraordinary presentations of GCT.

## FUNDING

This study was supported by JSPS KAKENHI Grant Number 22K16650.

## ETHICS STATEMENT

Approval of the research protocol: N/A.

Informed Consent: Obtained.

Registry and the Registration No. of the study/trial: N/A.

Animal Studies: N/A.

Research involving recombinant DNA: N/A.

## Data Availability

The data that support the findings of this study are available from the corresponding author upon reasonable request.
